# Isotopes, herds, and landscape management practices: New insights on early farming communities in the Serpis Valley (Eastern Iberian Peninsula)

**DOI:** 10.1371/journal.pone.0325137

**Published:** 2025-06-27

**Authors:** Vanessa Navarrete, Ismael Vaccaro, Pilar Escribá, Elena Grau, Oreto García-Puchol, Joan Bernabeu Aubán

**Affiliations:** 1 Institución Milà y Fontanals de investigación en Humanidades, Consejo Superior de Investigaciones Científicas, Barcelona, Spain; 2 Departament de Prehistòria, Arqueologia i Història Antiga, Grupo de investigación PREMEDOC, Universitat de València, Valencia, Spain; University of California Santa Cruz, UNITED STATES OF AMERICA

## Abstract

The establishment of the first livestock communities in the eastern Iberian Peninsula during the 6^th^ millennium cal BCE marked a significant transition in the region’s economic system. The research of early animal management practices provides crucial valuable understanding into feeding and pastoral strategies, revealing insights into the social organization of landscapes and their resources. Using stable carbon (δ^13^C) and nitrogen (δ^15^N) isotopic analyses of faunal remains this study investigates the interplay between environmental conditions, animal management practices, and dietary habits in Neolithic herds from Mas d’Is and Niuet sites in the Serpis Valley. The landscape of the area, characterized by Mediterranean forests and marshes, provided a variety of plant resources. Although most of the valley is covered by C_3_ types of plants, there were some clusters of C_4_ vegetation. The isotopic analyses prove that the local herds can be disaggregated in two groups: some animals with a diet based on C_3_, some feeding on C_4_ plants. These results reveal diverse feeding strategies and, by default, spatialized management practices. In addition, the animals presenting a C_3_ signature, have, as well, strong nitrogen values and marks on the bones that indicate they were part of the agricultural cycle as traction force. This complex organisational strategy, with two herds that show different levels of husbandry intensification, might reflect broader socio-economic systems, where the landscape may have been managed with a mosaic of different property regimes: where marginal areas might have been considered as communal or open-access resources, while more fertile areas nearby settlements, might have been used as crop fields, and individually or household managed. The integration of livestock into agricultural systems and the selective use of landscape resources highlight an adaptive and dynamic approach to animal husbandry in response to environmental and social factors during the Neolithic in the Serpis Valley.

## Introduction

The study of domestic fauna biochemistry and morphology remains offers an opportunity to understand long-gone, feeding practices and pastoral management strategies. These strategies, in turn, provide insight into the social organization of the landscape and its resources. The comprehension of key aspects of landscape usage such as herd management, intensity of environmental resources’ use, and settlement patterns contribute to the reconstruction of social practices during the establishment of the first livestock communities in the Iberian Peninsula during the 6^th^ millennium cal BCE. Understanding herd feeding practices is crucial for elucidating the relationship between sedentary societies and their environment, including the territorial distribution of grazing areas, spatial distribution of settlements, and access to distinct pasture areas [1–5]. Recent studies on the Neolithic in the northeast, south and west of the Iberian Peninsula, integrating δ^13^C and δ^15^N stable isotope analyses in zooarchaeological research, have revealed a high degree of variability in animal management practices, characterizing the access of flocks to distinct pasture areas and/or crop fields, and the use of fodder derived from crop by-products [6–11]. Nevertheless, this approach has not yet been applied to explaining early husbandry practices in the east of the Iberian Peninsula, one of the pioneering areas in the expansion of the Neolithic in Iberia.

In the archaeological context of the eastern Iberian Peninsula, the introduction of livestock practices inaugurated a distinctive phase that represented a new stage in territorial occupation. During the Neolithic, settlements were relocated from topographically dominant localization to the bottom of the valleys. In addition to these valley settlements, caves and shelters were used for specific economic and symbolic purposes (e.g., sheepfold caves and burials spaces), highlighting a multifaceted approach to land use [12–16]. As the Neolithic progressed, evidenced by archaeological record from the 4^th^ and 3^rd^ millennium BCE, sedentary settlements spread over large territories. Segmented ditches delimited open-air settlements and featured numerous structures, such as siles and pits [17–20]. By the 3^rd^ millennium cal BCE, a new cycle in the socio-ecological dynamics emerged, driven by demographic expansion, intensification of economic practices, and growing social complexity in the east of the Iberian Peninsula [21–25].

**Fig 1:**
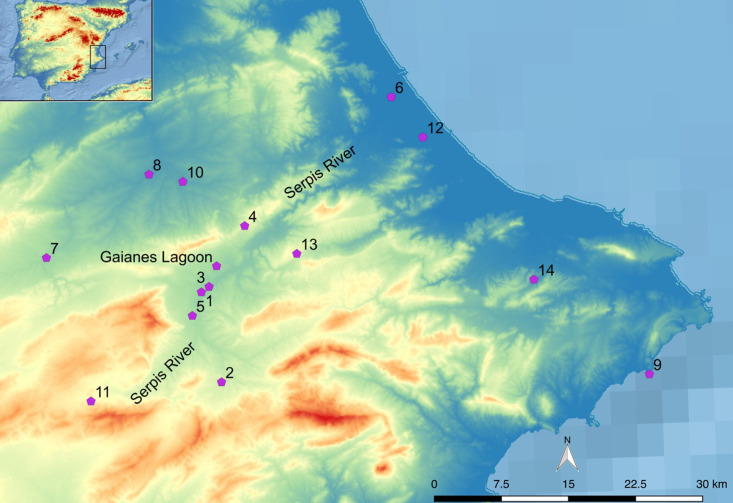
Archaeological sites mentioned in the text. 1-Niuet, 2- Mas d’Is, 3- Benàmer, 4-Cova de l’Or, 5-Jovades, 6-La Vital, 7-Arenal Costa, 8-Colata, 9-Cova Cendres, 10-Missena, 11-Abric de la Falguera, 12-Barranquet, 13-Cova d’en Pardo, 14-Randero. Gaianes’ Lagoon and Serpis River are also indicated.

The Serpis Valley, is revealed as one of the first areas that present evidence of the spread of the Neolithic in the Iberian Peninsula around the middle of the 6^th^ millennium cal BCE, offering onwards a rich and deep archaeological landscape (Fig 1). Numerous sites have been identified along the Serpis River and surrounding areas, from the early Neolithic to the Bronze Age. Archaeological excavations at sites such as Mas d’Is, Cova de l’Or, Abric de la Falguera, and Benàmer, which from the 6^th^ and 5^th^ millennia cal BCE [14,15,26,27], as well as Jovades, Niuet, and La Vital from the 4^th^ and 3^rd^ millennia cal BCE [17,20,21]; along with others such as Cova de les Cendres, Barranquet, En Pardo, Randero, Missena, Arenal de la Costa and Colata [28–33], have provided archaeological remains that offer a multifarious view of the economy and the environment throughout the Neolithic period.

From the beginning of the Neolithic, the main species of domesticated animals and plants were present in the region [34,35]. Agriculture and livestock breeding were the primary subsistence strategies for communities, with a relatively small contribution from hunting and gathering. The location of the settlements at the bottom of the valleys, surrounded by fertile soils, suggests the development of intensive agriculture with hoe in fields close to the inhabited centres [36–38].

Archaeobotanical data from several sites reveal a broad range of crops with a predominance of cereals. While wheat (*Triticum aestivum/durum*) and naked barley (*Hordeum vulgare var. nudum*), were the most common, hulled cereals like einkorn (*Triticum monococcum*), emmer (*Triticum dicoccum*) and hulled barley (*Hordeum vulgare subsp. vulgare*), also played a significant role in the cultivar portfolio [39]. A notable change occurred during the Neolithic was the reduction in the variety of cultivated cereals, that is, a process of relative specialization, which became particularly apparent starting on the 4^th^ millennium cal BCE. This process may have started in the 5^th^ millennium cal BCE, although data for this period is limited [40]. From the 4^th^ millennium cal BCE onwards, both wheat and naked barley, became the only cultivated cereal species [40]. Evidence of woodland clearcutting for larger-scale agriculture, combined with the prevalence of cereal-dominated crop assemblages, indicate that significant transformations were occurring in the economic system [37].

Husbandry practices were characterized by herds composed of caprines (*Ovis aries* and *Capra hircus*), cattle (*Bos taurus*) and pig (*Sus domesticus*) [35]. The regional archaeozoological record suggests a significant economic change by the end of the 4^th^ millennium cal BCE, marked by a notable increase in cattle remains and, compared to the 6^th^ millennium cal BCE data, a decrease in caprine remains. During the 6^th^ millennium cal BCE, cattle remains were represented in very low percentages at sites such as Cova de les Cendres with a 1% [41], Cova de l’Or with a 2% [42] and Cova la Sarsa with a 5.5% [43]. The limited data available from the 5^th^millennium cal BCE prevents direct comparisons of faunal remains between these periods. By the end of the 4^th^ millennium cal BCE, there was a progressive increase in cattle remains in the Serpis Valley, with sites like Jovades with a 15% [44] and Niuet with a 27% [45] showing significant growth. By the 3^rd^ millennium cal BCE, cattle represented over 35% of the domestic species in sites such as La Vital with a 35.5% [46] and Arenal de la Costa with a 36% [44]. Navarrete et al. [45], suggest that the increase in cattle remains in the Serpis Valley could be linked to early occupation of the bottom of valley and the development of the territory according to the open-air settlement structuration, benefiting cattle breeding, as these areas had favourable conditions such as vast plains, access to water, and abundant high-quality herbaceous grasses. These conditions were particularly prevalent in river and lake valleys, offering ample grazing areas for herds. Additionally, cattle could have acquired an additional economic significance due to their role in agricultural work (e.g., animal traction) and their contribution to milk and meat production. Slaughtering patterns and bone pathologies, such as joint injuries in distal extremities, observed at sites like Niuet [45] and Jovades [44], could be indicators of these changes.

These developments in livestock management must be contextualized within the broader ecological setting of the Serpis Valley, whose landscape may have played a key role in shaping agropastoral strategies. During the early Holocene, the Mediterranean landscape was dominated by deciduous forests and conifers, interspersed with open areas like pastures covered by herbaceous plants [47]. In particular, the Serpis Valley featured Mediterranean forests dominated by holm oaks and pines, with riparian zones along the Serpis River characterized by poplar and willow groves, as well as reedbeds [48,49]. Within this valley is the Gaianes Lagoon, a wetland with a rich and biodiverse ecosystem (Fig 1). The vegetation in this area is typical of wetlands and marshes, comprising a wide variety of plant species adapted to flooding and high humidity conditions. The lacustrine landscape includes reeds and rushes, aquatic vegetation like duckweed and pondweeds, marsh plants such as sawgrass and club-rushes, riparian vegetation including willows and alders, halophytic plants like glassworts and sea lavenders, and emergent vegetation such as sea milkwort [50].

By combining carbon (δ^13^C) and nitrogen (δ^15^N) stable isotope analysis of bulk collagen with archaeozoological data, the study aims to investigate early husbandry practices in the Serpis Valley, a key area for understanding the spread of the Neolithic in the Iberian Peninsula. This study characterises management strategies by identifying grazing patterns and foddering practices. Particular attention is given to how these practices were influenced by the environmental context of the Serpis Valley, including the availability of fertile valley bottoms, riparian ecosystems, and wetland areas. Additionally, this study contributes to a broader understanding of landscape use and subsistence strategies during the Neolithic. To address these objectives, two Neolithic sites from the Serpis Valley -Mas d’Is and Niuet- have been selected, as they provide a representative sequence for the regional Neolithic occupation.

## Archaeological sites

### Mas d’Is site

**Fig 2:**
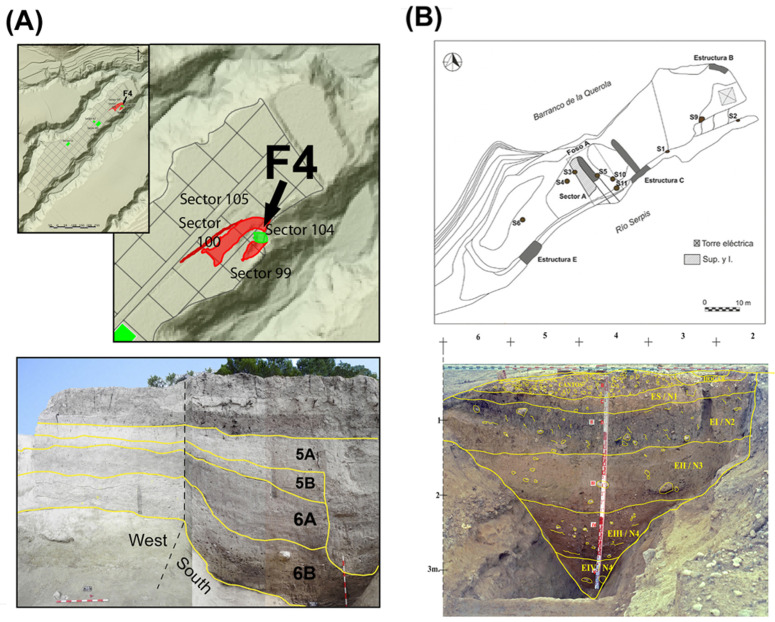
Mas d’Is and Niuet sites. **A)** Above: plan view of the Mas d’Is site, showing the location of ditch F4. Below: stratigraphy of ditch F4. **B)** Above: plan view of the Niuet site, showing the location of Sector A. Below: stratigraphy of Sector A.

The Mas d’Is is an open-air site located within the municipality of Penàguila (Alicante) at 600 metres above sea level (masl), in the valley bottom of the Serpis Valley. It is located at the head of the Penàguila River, a tributary of the Serpis River, the main watercourse in this region (Fig 2A). The site covers a large area with documented occupations from the early Neolithic to the Bronze Age, including some of the earliest radiocarbon dates connected to the Neolithic in the Iberian Peninsula (Table 1). The first Neolithic occupations are documented at the levels associated with the habitat structures of sector 80 [51] and at the base of the ditch (F4) [26]. F4 presents the most extended stratigraphic sequence within the site, and archaeological research has identified eight archaeological phases (Fig 2A).

**Table 1:** Radiocarbon (^14^C) dates from the Mas d’Is and Niuet sites.

Site	Sector	Phase	^14^C yr BP	Material	^14^C yr cal BCE 95% 2σ	Reference	Lab Code
Mas d’Is	F4	5	5400 ± 35	*Hordeum* sp.	4341−4065	[57]	CNA482811
Mas d’Is	F4	5	5492 ± 36	Cereal Seed	4444−4255	[57]	CNA482911
Mas d’Is	F4	5	5590 ± 40	*Triticum aest./durum*	4498−4347	[12]	Beta171908
Mas d’Is	F4	6	6030 ± 30	*Bos taurus*	5007−4805	[12]	Beta331018
Mas d’Is	F4	6	6120 ± 30	*Cervus elaphus*	5209−4947	[57]	Beta527801
Mas d’Is	F4	6	6140 ± 30	*Bos taurus*	5210−4997	[58]	Beta331019
Mas d’Is	F4	6	6160 ± 40	*Quercus* sp.	5216−4994	[12]	Beta162093
Niuet	Sector A	N1	4170 ± 30	*Bos taurus*	2883−2632	[45]	Beta527806
Niuet	Sector A	N2	4200 ± 30	*Ovis aries*	2895−2671	[45]	Beta527805
Niuet	Sector A	N2	4460 ± 60	*Quercus* sp.	3351−2929	[21]	Beta75223
Niuet	Sector A	N3	4375 ± 54	*Bos taurus*	3326−2891	[57]	AA72171
Niuet	Sector A	N3	4410 ± 30	*Ovis aries*	3315−2916	[45]	Beta527804
Niuet	Sector A	N3	4490 ± 60	Charcoal	3366−2936	[21]	Beta75222
Niuet	Sector A	N4	4460 ± 30	*Bos taurus*	3337−3021	[45]	Beta527803
Niuet	Sector A	N4	4404 ± 32	*Ovis aries*	3314−2912	[45]	CIRAM9702

Phases 8 to 5 correspond to the Neolithic IA, IB and IIA in the regional sequence (from c. 5600 to 4850 and to 4550 to 4250 cal BCE [52]). Although Phases 8 and 7, represent the early Neolithic (*Impressa* and Cardial phases [53]), the scarcity of faunal remains limits their relevance for the present study. Instead, the focus is on Phase 6 and 5, which offer valuable insights into herd management strategies and their socioeconomic implications. Phase 6, associated with the Neolithic IB, dated between 5250-4850 cal BCE [52], is characterized by incised-impressed ceramics. Phase 5 aligns with the regional Neolithic IIA, dated between 4550-4250 cal BCE [52]. Subsequently, various episodes concluded the prehistoric sequence of the site at a time parallel to the Bronze Age [12,51].

The subsistence economy during Phase 6 and 5 appears to have been primarily farming practices, complemented by a broad exploitation of forest resources. Despite the poor preservation of organic matter, the zooarchaeological results show that in both phases domestic species (caprine, pig and cattle) predominated over wild species (deer and rabbit), indicating a husbandry practice. Although the percentage of remains is limited, Phase 6 shows a prevalence of cattle remains, while Phase 5 is dominated by pig remains, which may suggest a change in herd composition strategies. Evidence points to a focus on meat production in both phases, as evidenced by the predominance of adult individuals between 18–24 months old among recovered faunal remains. This age range aligns with optimal meat yield before maintenance cost increase (S1 Table). Archaeobotanical data show the predominance of cereals in both phases. Phase 6 includes hulled barley, wheat and einkorn, and Phase 5 naked barley and wheat [39]*.*

Anthracological analyses indicate a diverse use of forest resources, with the predominance of *Quercus* (*Evergreen Quercus* and *Quercus* sp.) throughout the archaeological sequence. In addition, in Phase 6, remains of *Juniperus* sp., and in Phase 5 remains of cf. *Clematis* sp., *Coniferae, Juniperus* sp., *Leguminosae, Pinus halepensis, Pinus* sp. and Monocotyledoneae [54] (S2 Table) have been recovered. This diversity in Phase 5 underscores the interaction between environmental resources and farming economies, suggesting the use of surrounding forests for wood, fuel, and possible complementary food sources.

Conventional radiocarbon date along with calibrated radiocarbon ages using OxCal 4.4.4 software of the study phases of the Mas d’Is and Niuet sites (IntCal20.14c [55,56]).

### Niuet site

Niuet is an open-air site located on the left bank of the Serpis River, in the valley bottom of the Alcoi Valleys (Serpis Valley), at 340 masl, within the municipality of l’Alqueria d’Asnar (Alicante) (Fig 2B). Situated on a terrace in an interfluve, it is bordered by the Serpis River on one side and the Querola ravine (Barranquet de Vargas) on the other. Archaeological works have identified three sectors: western, eastern and central. Sector A, in the central area, is the richest archaeologically, covering 41m^2^. Five stratigraphic levels have been documented, with the four lower levels (II to V) corresponding to the filling of a ditch with a V-shaped cross-section, measuring 5.5 m in width and 2.4 m in depth [21] (Fig 2B). All ^14^C dates were obtained from Sector A, placing the Niuet site sequence between the end of the 4^th^ millennium and the beginning of the 3rd millennium cal BCE. Phase 2, including Phases N3 and N4 (levels: V, IV, III, IId, IIc), span from the end of the 4th millennium to the beginning of the 3rd millennium cal BCE (c. 3300-2900 cal BCE), while Phase 1, includes Phases N1 and N2 (levels: IIb, IIa, I, ES) and corresponds to the beginning of the 3^rd^ millennium cal BCE (c. 2900-2600 cal BCE) [21,45] (Table 1). These chronological sequences provide a framework for examining shifts in economic practices and their implications for herd management and resource exploitation.

The economic practices at the Niuet site reflect a diversified subsistence strategy, where farming was complemented by the exploitation of forest and riparian resources. Livestock consisted of mixed and multipurpose herds, with caprines predominating across all phases analysed. During this period (Phases 1 and 2) cattle becomes increasingly more significant, eventually reaching a third of the herd in both phases. The primary production focus of livestock was meat, but the herds were likely used for multiple purposes, including dairy production and bovine strength for agricultural tasks [45]. This hypothesis is supported by the recovery of part of a pottery recipient with holes that could correspond to a cheese maker [21] and evidence of traction work, possible agricultural work, related pathologies in cattle [45]. Additionally, the analysis of sheep size has yielded new insights into the discussion of animal size reduction at the beginning of the 3rd millennium cal BCE [35,45]. Archaeobotanical data show the presence of cereals such as wheat and leguminous such as grass pea.

Anthracological evidence reflects to the extensive exploitation of forest and riparian vegetation, with *Evergreen* Quercus dominating throughout the Neolithic sequence, complemented by shrub taxa such as pistacia or leguminosae. Riparian vegetation, including *Fraxinus oxyhylla*, *Salix* sp., *Populus nigre*, *Crataegus* sp., and *Laurus nobilis*, reflect the use of nearby water-associated resources [59].

## Materials and methods

### Sample selection of isotopic analysis

No permits were required for the described study, which complied with all relevant regulations.

A total of 66 faunal samples from Mas d’Is and Niuet sites were selected for stable isotope analysis of carbon and nitrogen in bone collagen. To establish the local δ^13^C and δ^15^N isotopic baselines for wild and domestic mammals’ species, we selected a range of herbivores, omnivores and carnivores represented at the archaeological sites. To ensure representation from different individuals, the diaphysis of long bones from the same skeletal element and laterality were analysed for each animal species and stratigraphic level. When long bones were unavailable, mandible and phalanx were selected.

From the F4 of Mas d’Is site, 18 samples were selected: 12 from Phase 6 (5250-4850 cal BCE), and 6 from Phase 5 (4550-4250 cal BCE). The samples include domestic herbivores (*Capra hircus* Phase 6 n = 2; *Bos taurus* Phase 6 n = 8, Phase 5 n = 1; *Ovis aries* Phase 5 n = 1), domestic omnivores (*Sus domesticus* Phase 5 n = 2) and wild herbivores (*Cervus elaphus* Phase 6 n = 2, Phase 5 n = 2).

From the Sector A of Niuet site, 48 samples were selected. 8 samples from Phase 2 (c. 3300-2900 cal BCE), and 40 samples from Phase 1 (c. 2900-2600 cal BCE). Domestic herbivores (*Capra hircus* Phase 2 n = 2, Phase 1 n = 8; *Ovis aries* Phase 2 n = 2, Phase 1 n = 9; *Bos taurus* Phase 2 n = 1, Phase 1 n = 13), domestic omnivores (*Sus domesticus* Phase 2 n = 3, Phase 1 n = 5), wild herbivores (*Cervus elaphus* Phase 1 n = 2; *Oryctolagus cunniculus* Phase 1 n = 2), and carnivores (*Canis familiaris* Phase 1 n = 1) have been included in this study.

The limited number of samples from Phase 5 of Mas d’Is site (n = 6) and from the Phase 2 at Niuet site (n = 8) is due to the poor preservation of organic remains at these sites. Even so, these samples come from adult individuals of animal species and provide valuable information for the analysed periods, contributing to the isotopic baseline of the eastern Iberian Peninsula.

### Collagen extraction and δ^13^C and δ^15^N stable isotope analysis

Sample selection was conducted at the University of Valencia (Spain). Collagen extraction took place at the HERCULES Laboratory at the University of Évora (Portugal). Samples (ca. 200–300 mg) were cleaned to remove contaminants using a DREMEL tool. Collagen extraction followed the modified method of Longin [60]. The bone samples were demineralized in 10 ml of 0.5 M HCl at 4°C for approximately 14 days. Afterward, the samples were cleaned with ultrapure water until neutral and immersed in 0.125 M NaOH for 20 hours at room temperature. Subsequently, they were gelatinized in 0.01 M HCl at 70°C for 48 hours. To remove impurities, the liquid fraction was filtered using Ezee‑Filter™, and the solubilized collagen was freeze-dried for 48 hours.

Collagen samples (0.3 mg) were analysed using a Thermo Flash 1112 elemental analyser (EA) coupled to a Thermo Delta V Advantage isotope ratio mass spectrometer (IRMS) with a Conflo III interface, at the Institute of Science and Technology of the Autonomous University of Barcelona (ICTA‑UAB). The average analytical error was less than 0.2‰ (1σ), as determined from duplicate analyses of δ^13^C and δ^15^N. The international laboratory standard IAEA 600 (caffeine) was used for control. Vienna PeeDee Belemnite (V‑PDB) was used for δ^13^C, while N2 air (AIR) served as standard for δ^15^N. A one-way ANOVA (α = 0.05) was used to compare δ13C values among wild and domestic herbivores and omnivores, following verification of normal distribution with the Shapiro–Wilk test (α = 0.05). The null hypothesis of normal distribution was rejected for the δ^15^N values, so these were analysed using the Kruskal–Wallis test (α = 0.05). All statistical analyses were conducted using PAST 4.11 [61].

### δ^13^C and δ^15^N isotope analysis of bulk collagen for animal dietary reconstruction

Bulk collagen carbon (^13^C/^12^C) and nitrogen (^15^N/^14^N) stable isotopes provide valuable insights into early pastoral management strategies by characterizing the contributions of animal and plant macronutrients to animal diets [52–64]. Collagen, the primary protein in bone, reflects an individual’s diet over an extended period of time (approximately 5–15 years old) through its carbon and nitrogen isotope composition [65].

Stable carbon isotopes vary significantly among terrestrial, marine, and estuarine ecosystems, and between plants with different photosynthetic pathways (e.g., C_3_ and C_4_ plants) [66–68]. These variations are also influenced by environmental factors such as water availability, ambient temperature, and CO_2_ concentrations [69–72]. According to a carbon isotope fractionation of ~5‰ between whole plants and consumer’s bone collagen [62] and a correction of ~ + 1.5‰ for the fossil fuel effect in pre-industrial ecosystems [73,74], δ^13^C values around or above than −19‰ in herbivorous species could indicate the consumption of drought-resistant or dry vegetation, such as shrubs. These plants often have higher δ^13^C values due to water stress and reduced photosynthetic discrimination against ^13^C. On the other hand, values greater than −16‰ would suggest the consumption of C_4_ plants, most common in water saturated environments [72,75].

Nitrogen isotopes derive exclusively from dietary protein, with δ^15^N values typically indicating the trophic level of the food source due to predictable isotopic fractionations (approximately +3‰ to +6‰) throughout the food web [62,68,76]. Furthermore, increases of bulk collagen δ^15^N values in humans and animals in agricultural settings can reflect variations in δ^15^N abundance in plant-soil systems influenced by management practices, such as the use of animal fertilizers [77,78].

## Results

### Collagen quality

The results of stable isotope analyses of δ^13^C and δ^15^N in bulk bone collagen are summarized in Table 2.

**Table 2:** Results from δ^13^C and δ^15^N isotopic analysis conducted on faunal samples from the Mas d’Is and Niuet sites.

Site	Specimen ID	Phase	Layer	Specie ID	Skeleton part	%yield	%C	%N	δ^13^C	δ^15^N	C:N
Mas d’Is	1	6	104393	*Bos taurus*	Metatarsal	0.6	17.8	5.8	−20.5	4.9	3.6
Mas d’Is	4	6	105611	*Bos taurus*	Mandible	4.8	18.7	6.2	−17.3	5.6	3.5
Mas d’Is	5	6	105619	*Bos taurus*	Metacarpal	4.5	16.9	5.7	−17.8	6.2	3.5
Mas d’Is	6	6	104812	*Bos taurus*	Metatarsal	4.3	18.2	6.3	−14.7	4.8	3.4
Mas d’Is	11	6	104813	*Bos taurus*	Metacarpal	4.0	19.2	6.7	−17.7	4.1	3.3
Mas d’Is	2	6	105612	*Capra hircus*	Mandible	4.1	40.2	13.9	−19.9	6.1	3.4
Mas d’Is	8	6	104819	*Cervus elaphus*	Phalanx 2	3.6	41.5	14.2	−20.8	3.5	3.4
Mas d’Is	13	5	105605	*Cervus elaphus*	Phalanx 2	4.2	31.4	10.6	−20.3	4.0	3.4
Mas d’Is	15	5	105604	*Sus domesticus*	Talus	3.9	21.1	7.3	−19.7	3.7	3.4
Mas d’Is	17	5	104928	*Cervus elaphus*	Radius	3.7	34.5	12.1	−20.2	3.5	3.3
Mas d’Is	18	5	104939	*Bos taurus*	Tibia	3.7	19.2	6.7	−16.9	4.5	3.4
Niuet	6	1	A3-6-S	*Bos taurus*	Tibia	2.4	31.8	11.2	−14.1	5.2	3.3
Niuet	8	1	b4-7–9-I	*Bos taurus*	Tibia	1.6	41.7	15.3	−18.1	6.0	3.2
Niuet	14	1	A4-12-I	*Bos taurus*	Humerus	2.7	35.7	12.2	−20.4	10.9	3.4
Niuet	15	1	b4-12-I	*Bos taurus*	Metacarpal	4.4	40.8	14.8	−19.6	4.9	3.2
Niuet	16	1	b4-12-I	*Bos taurus*	Metacarpal	3.8	33.3	11.2	−20.3	3.4	3.5
Niuet	17	1	D-1-S	*Bos taurus*	Tibia	1.6	40.2	14.6	−18.2	5.8	3.2
Niuet	19	1	B4-20-II	*Bos taurus*	Tibia	1.0	38.7	13.7	−14.3	5.7	3.3
Niuet	21	1	a5-11-I	*Bos taurus*	Metacarpal	4.7	23.9	8.1	−20.0	8.3	3.5
Niuet	25	1	a3-11-I	*Bos taurus*	Radius	9.0	28.4	10.0	−17.4	4.8	3.3
Niuet	26	1	C4-20-II	*Bos taurus*	Femur	2.4	40.6	14.5	−19.0	5.9	3.3
Niuet	27	1	a4-19-II	*Bos taurus*	Humerus	1.8	41.3	14.8	−14.4	5.0	3.3
Niuet	28	1	A4-7-I	*Bos taurus*	Metacarpal	5.7	41.2	14.7	−20.0	3.9	3.3
Niuet	42	1	O3-2-I	*Bos taurus*	Tibia	3.9	41.9	14.7	−19.1	7.4	3.3
Niuet	1	1	A5-10-I	*Capra hircus*	Metatarsal	5.3	37.8	13.5	−20.5	5.6	3.3
Niuet	13	1	A4-8-I	*Capra hircus*	Metatarsal	1.6	40.7	14.5	−19.6	5.9	3.3
Niuet	22	1	a5-9-I	*Capra hircus*	Metatarsal	3.1	41.1	14.7	−20.4	5.4	3.3
Niuet	35	1	h/i7-2-I	*Capra hircus*	Humerus	1.2	41.3	15.1	−19.9	4.1	3.2
Niuet	38	1	b4-12-I	*Capra hircus*	Metacarpal	2.4	40.3	14.2	−18.9	5.4	3.3
Niuet	40	1	a4-15-II	*Capra hircus*	Metatarsal	1.6	41.2	14.8	−19.4	4.3	3.3
Niuet	10	1	a5-10-I	*Cervus elaphus*	Tibia	2.0	36.2	13.0	−19.8	4.0	3.2
Niuet	32	1	II	*Cervus elaphus*	Tibia	2.2	41.1	14.6	−20.1	4.5	3.3
Niuet	9	1	H5-19-II	*Oryctolagus cunniculus*	Calcaneous	1.3	41.1	15.0	−21.1	5.3	3.2
Niuet	2	1	a5-10-I	*Ovis aries*	Metatarsal	2.3	39.6	14.1	−19.7	5.5	3.3
Niuet	3	1	A2-2-S	*Ovis aries*	Tibia	5.7	39.2	14.0	−19.1	6.1	3.3
Niuet	7	1	B4-22-S	*Ovis aries*	Humerus	2.7	41.5	14.9	−20.1	6.5	3.3
Niuet	30	1	a3-12-I	*Ovis aries*	Radius	4.1	40.3	14.7	−19.7	7.2	3.2
Niuet	31	1	A3-8–9-I	*Ovis aries*	Metacarpal	2.3	36.1	12.7	−20.0	2.9	3.3
Niuet	34	1	B4-12-I	*Ovis aries*	Humerus	1.8	41.6	14.6	−19.5	5.3	3.3
Niuet	37	1	H5-22-II	*Ovis aries*	Humerus	2.2	40.8	14.9	−20.4	5.0	3.2
Niuet	41	1	c5-19-II	*Ovis aries*	Metatarsal	1.2	41.6	14.8	−20.5	6.8	3.3
Niuet	47	1	A5-22-II	*Ovis aries*	Mandible	3.9	41.0	14.5	−20.7	7.0	3.3
Niuet	4	1	a3-4-S	*Sus domesticus*	Humerus	6.4	39.2	14.2	−21.0	5.3	3.2
Niuet	20	1	C5-18-II	*Sus domesticus*	Humerus	1.7	41.0	14.8	−19.8	8.5	3.2
Niuet	45	1	a4-22-II	*Sus domesticus*	Femur	1.3	43.1	15.2	−20.0	5.4	3.3
Niuet	33	1	II	*Sus domesticus*	Humerus	2.2	40.8	14.7	−20.2	5.4	3.2
Niuet	43	2	a4-27-III	*Bos taurus*	Calcaneous	3.6	40.6	14.2	−19.2	5.2	3.3
Niuet	24	2	A4-27-III	*Capra hircus*	Mandible	4.9	40.3	14.5	−19.3	6.9	3.2
Niuet	48	2	a4-28-III	*Capra hircus*	Mandible	10.2	35.5	12.3	−18.6	5.9	3.4
Niuet	46	2	c5-22-III	*Ovis aries*	Humerus	1.5	35.9	12.6	−20.4	5.8	3.3
Niuet	23	2	A4-5–29-III	*Sus domesticus*	Skill	4.0	39.7	14.2	−20.2	7.2	3.3
Niuet	44	2	ABC4–5-III	*Sus domesticus*	Femur	2.3	41.4	14.8	−21.0	6.0	3.3

At the Mas d’Is site, collagen was successfully extracted from 11 out of 18 samples (61%). Collagen yields ranged from 0.6 to 5%. Phase 5 has the lowest levels of collagen yields. The C% and N% values ranged from 17 to 41%, and from 6 to 14%, respectively, with C:N ratios between 3.3 to 3.6, consistent with the preserved collagen values proposed by DeNiro [79] and Van Klinken [76]. Cut-off values of 13% for C% and 4.8% for N% were applied, as recommended by Ambrose [81].

At the Niuet site, collagen was successfully extracted from 41 out of 48 samples (89%). Collagen yields varied widely, ranging from 1 to 13%, with no significant differences across species or Phases. The C% and N% values ranged from 24 to 43%, and from 8 to 15%, respectively, with C:N ratios between 3.2 to 3.5. These results are consistent with the values proposed by DeNiro [79] and Van Klinken [80], and the cut-off values of 13% for C% and 4.8% for N% were also applied, as recommended by Ambrose [81].

### δ^13^C and δ^15^N values at the Mas d’Is site

**Fig 3:**
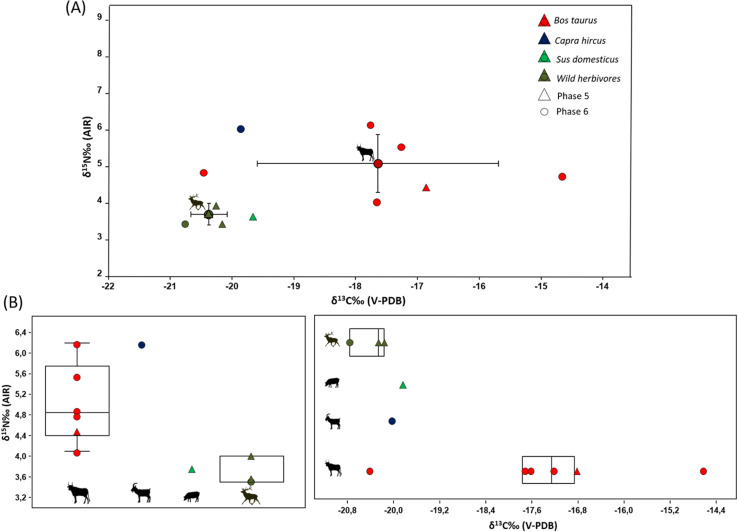
δ^13^C and δ^15^N isotopic analysis results from Mas d’Is site. (A) Bone collagen δ^13^C and δ^15^N values compared to the mean δ^13^C and δ^15^N values ±1σ s.d for each taxon from the Mas d’Is site. (B) Box plot δ^13^C and δ^15^N values form faunal samples from the Mas d’Is site.

At the Mas d’Is site, the average δ^13^C value was ‑18.7 ± 1.9‰ and the average δ^15^N value was 4.6 ± 1.0‰ (n = 11). Group-specific values were as follows: wild herbivores had δ^13^C x̄ = ‑20.4 ± 0.3‰, δ^15^N x̄ = 3.7 ± 0.3‰ (n = 3), domestic herbivores had δ13C x̄ = ‑17.8 ± 1.9‰, δ^15^N x̄ = 5.2 ± 0.8‰ (n = 7) and domestic omnivore had δ^13^C = ‑19.7‰, δ15N = 3.7‰ (n = 1). From Phase 5, collagen was obtained from deer (δ^13^C x̄ = −20.3 ± 0.1‰, δ^15^N x̄ = 3.7 ± 0.4‰, n = 2), pig (−19.7‰, n = 1) and cattle (−16.9‰, n = 1). In Phase 6, collagen was obtained from cattle (δ^13^C x̄ = −17.6 ± 2.0‰, δ^15^N x̄ = 5.1 ± 0.8‰, n = 5), goat (−19.9‰) and deer (−20.8‰). Cattle in Phase 6 exhibited significant variability of δ^13^C values, ranging from −20.5‰ to −14.7‰, documenting a −5.7‰ intra-range variability. When comparing δ^15^N values, goat showed the highest enrichment of δ^15^N among domestic herds compared to wild herbivores (2.4‰), followed by cattle in Phase 6 (1.4‰) and in Phase 5 (0.8‰). The pig shows the same δ^15^N value as average wild herbivores (Fig 3).

### δ^13^C and δ^15^N values at the Niuet site

At the Niuet site, the average δ^13^C value was ‑19.4 ± 1.6‰ (Phase 2, n = 6; Phase 1, n = 35). Wild herbivores, represented only in Phase 1, had an average δ^13^C value of −20.4 ± 0.7‰ (n = 3). No significant statistical differences were found between domestic herbivores and domestic omnivores across the phases. Domestic herbivores had an average δ^13^C values of −19.1 ± 1.8‰ (Phase 2 = −20.4‰ to −18.6‰ n = 4; Phase 1 = −20.7‰ to −14.1‰ n = 31), while domestic omnivores averaged −20.4 ± 0.5‰ (Phase 2 = −21.0‰ to −20.2‰, n = 2; Phase 1 = −21.0‰ to −19.8‰, n = 4). In Phase 2, the intra-range specie δ^13^C variability was noted in goat (−0.7‰) and pig (−0.8‰). In Phase 1, cattle showed the greatest intra-range specie δ^13^C variability (−6.3‰) compared to goat (−1.6‰), sheep (−1.6‰) and pig (−1.2‰), with significant differences observed between cattle and sheep (р = 0.03).

**Fig 4:**
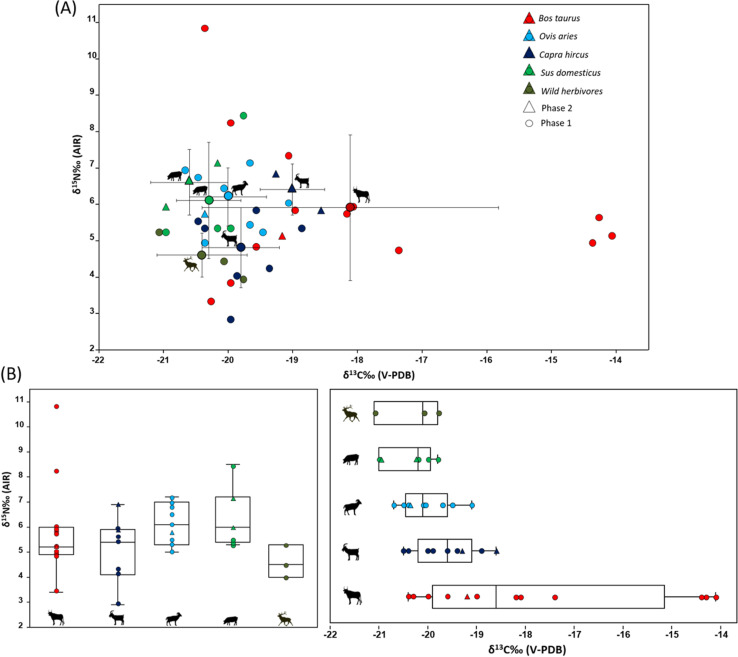
δ^13^C and δ^15^N isotopic analysis results from Niuet site. (A) Bone collagen δ^13^C and δ^15^N values compared to the mean δ^13^C and δ^15^N values ±1σ s.d for each taxon from the Niuet site. (B) Box plot δ^13^C and δ^15^N values form faunal samples from the Niuet site.

The average δ^15^N value for the Niuet site was 4.6 ± 1.0‰ (Phase 2, n = 6; Phase 1, n = 35). Wild herbivores had an average δ^15^N values of 4.6 ± 0.6‰ (Phase 1, n = 3). For domestic herbivores, the average δ^15^N value was 5.7 ± 1.5‰ (Phase 2, n = 4; Phase 1, n = 31), and for domestic omnivores, it was 6.3 ± 1.3‰ (Phase 2, n = 2; Phase 1, n = 4). Among domesticates, there were no significant statistical differences in δ^15^N values. Comparing domestic herds to wild herbivores in Phase 2, pig showed the highest δ^15^N-enrichment (2.0‰), followed by goat (1.8‰), sheep (1.2‰) and cattle (0.6‰). In Phase 1, pig and sheep showed the highest δ^15^N-enrichment (1.6‰), followed by cattle (1.3‰), and goats (0.2‰), with cattle reaching a peak δ^15^N value of 10.9‰. Among caprines, in Phase 2 the δ^15^N intra-range variability is 1.1‰, specifically 1‰ among goats, there is no data on sheep because there is only one individual analysed. And in Phase 1 the δ^15^N intra-range variability is 4.3‰, specifically 4.35‰ among sheep and 1.8‰ among goats (Fig 4).

## Discussion

### Pig, caprine and cattle herd management strategies in the Serpis Valley

The isotopic variability in δ^13^C and δ^15^N values from Mas d’Is and Niuet sites suggests that herds had access to a diverse range of plant resources, reflecting specific feeding strategies employed by Neolithic communities in the Serpis Valley.

In the study area, C_3_ plants are predominantly expected, with δ^13^C variations up to 4‰ throughout the seasonal cycle, peaking in summer and dipping in winter depending on water stress [70,72,82–84]. The δ^13^C values from the faunal remains of Mas d’Is and Niuet sites suggest that majority of the protein in these animals was obtained from a terrestrial food web based either on C_3_ or C_4_ plants. C_3_ values would be characterized by plants from open areas, while the higher δ^13^C values observed in cattle in Mas d’Is site (−14.7‰) and Niuet site (−14.1‰, −14.3‰ and −14.4‰) indicate the presence of environments dominated by C_4_ plants [85]. The selective targeting of grazing areas, particularly those adapted to wetland environments, may be the cause of high δ^13^C values [86]. These aquatic or semi-aquatic C_4_ species can thrive in environments with variable moisture levels, contributing to the overall plant diversity available to grazing animals [87]. The presence of such plants in the diets of cattle could further explain the elevated δ^13^C values. In the Serpis Valley, the *Cyperaceae* family, which includes plants located in wetland and marshes contexts like *Juncus* sp., *Carex* sp., *Scirpus* sp. and *Schoenoplectus* sp., could contribute to the C_4_ signal. *Cyperaceae* plants are adapted to saturated soils and flooded conditions, and while they are not grasses, they play a crucial role in wet ecosystems, and provide important habitats for various species of fauna. These Cyperaceous plants might have been present during the Neolithic along the Serpis River and more abundant in wetland areas like the Gaianes Lagoon, and their recurrent consumption could be reflected in the C_4_ signal observed in the δ^13^C values.

Documented evidence of C_4_ plants in the Iberian Peninsula during the Neolithic is scarce, with evidence primarily found at archaeological sites such as Costamar (Castellón, Spain) and La Vital (Gandia, Spain), both in eastern Iberia [88,89].

#### Pig management.

Pigs show δ^13^C values from open C_3_ environments at both the Mas d’Is and Niuet sites. At the Mas d’Is site, the δ^15^N value of the single individual analysed is similar to that of wild herbivores, suggesting an herbivorous diet without agricultural surplus contributions. At the Niuet site, pigs exhibit the least variability in δ^13^C values among the analysed herds, suggesting access to a narrower range of plant resources. The differences in δ^15^N values are observed between pigs from Phase 2 and Phase 1. In Phase 2, a greater enrichment of δ^15^N values is observed, possibly indicating a higher intake of protein in the pigs’ diet. This may suggest that pigs had access to protein sources not directly related to plant consumption, such as animal by-products or agricultural by-products. In contrast, pigs from Phase 1 show lower δ^15^N values, similar to wild herbivores, except for one individual displaying significantly enriched δ^15^N values (8.5‰), which could be indicative of a distinct dietary strategy or an unusual feeding pattern. The variability in δ^15^N values could reflect differences in the amount of animal protein in pigs’ diet [90] Pigs, being omnivorous, could have supplemented their plant-based diet with various protein sources, such as worms, insects, small animals, or human food waste. The lower δ^15^N values, closer to those of wild herbivores, suggest a diet richer in plant products, possibly indicating that pigs were managed in extensive systems in semi-free-range conditions. In contrast, the higher δ^15^N values in some pigs could be related to an increased intake of protein, possibly from waste disposal sites or access to agricultural by-products [2,6,91]. This interpretation has also been proposed for the Pyrenees [7,11], the Mediterranean areas [6,9] and the Atlantic areas of the Iberian Peninsula [8]. This dietary flexibility would have allowed pigs to take advantage of various food resources in human-dominated landscapes, where waste from daily activities, including animal processing and crop production, provided an accessible source of nutrition. Such enrichment in δ^15^N values may reflect the early stages of a more intensive pig-rearing strategy, where pigs were increasingly integrated into the livestock economy through the use of human-generated food surpluses and proximity to settlements, perhaps even stabled.

#### Caprine management.

The variation in δ^13^C values among the caprine herds at Mas d’Is and Niuet sites suggests a diet of open C_3_ plants, typical of environments dominated by trees and scrubs. This is a common dietary pattern for animals grazing in open areas, where C_3_ plants predominate. At Mas d’Is site, the isotopic analysis of a single goat revealed ^15^N-enriched values (2.4‰) compared to wild animals, which could suggest that this individual had access to fields or was provided with surplus crops as forage. At Niuet site, caprines from chronological Phases 2 and 1 show very similar δ^13^C isotopic signatures (difference δ^13^C 0,5‰ between phases), suggesting a relatively stable environment across the 4^th^ millennium and the beginning of the 3^rd^ millennium cal BCE, as also reflected in the anthracological analyses of the site [60]. δ^15^N values show high variability among caprines, mainly in Phase 1. In Phase 2, goats show more δ^15^N enriched values than sheep, although the intra-range variability amplitude is 1.1‰. In contrast, in Phase 1, goats show values similar to wild herbivores, unlike sheep, which show an intra-range variability of 4.3‰, and with an ^15^N-enrichment of 0,7‰ over goats. The δ^15^N values observed in the goats of Phase 1, point to grazing in areas with minimal human impact, such as soils not used for agriculture or arid areas. Is possible that goats were managed in environments outside cultivated areas or areas less suitable for intensive farming. In addition, the difference with some of the sheep’s diets could be affected by the type of diet of goats, which are browsers, also feeding on branches and bushes. Sheep, in Phase 1, in general, show higher δ^15^N values than goats. This enrichment could correspond to a more selective feeding, based on different grazing areas, access to fields or on the supply of agricultural surplus. The elevated δ^15^N values in some sheep samples could also be linked to the physiological needs of neonatal and infantile individuals under 3 months of age, as feeding practices are crucial for the nutritional well-being of the mother, which is essential for the survival of lambs and to ensure sufficient milk production [92]. In Phase 1, the recovery of a cheese maker fragment, a ceramic vessel with small perforations in the walls, provides additional evidence to support the use of milk and the consumption of dairy products at the Niuet site [21]. The caprines feeding practices at Mas d’Is and Niuet sites indicate early forms of controlled herding and the integration of livestock into agricultural activities and environmental management.

#### Cattle management.

Cattle exhibit significant variability in δ^13^C values at both sites, suggesting that different individuals consumed plants with different isotopic compositions. This variation may reflect differing animal management practices within the same archaeological site and concerning the same animal species. The δ^13^C values for the analysed specimens range from −20.5‰ to −16.9‰ and −14.7‰ at Mas d’Is site, and from −20.4‰ to −17.4‰ and from −14.4‰ to −14.1‰ at Niuet site. These results suggest that while some cattle consumed C_3_ plants, others consumed C_4_ plants. This feeding strategy appears to have been consistently applied at both sites, regardless of their location and chronology, reflecting a continuous compartmentalized management approach of the landscape throughout the Neolithic sequence in the Serpis Valley. Regarding the δ^15^N values, cattle from Mas d’Is site show a ^15^N-enriched by 1.4‰ in Phase 6, and 0.8 ‰ in Phase 5, and Niuet site show a ^15^N-enriched by 0.6‰ in Phase 2, and 1.3‰ in Phase 1, compared to wild herbivores. The diachronic analysis of the Mas d’Is and Niuet sites shows differences in δ^15^N values, which could reflect a change in the intensity of cattle management over time. At Mas d’Is site, cattle exhibit δ^15^N values reaching 6.2‰ with a variability range of 2.1‰. In contrast, at Niuet site, δ^15^N values reached 10.9‰, with a variability range of 7.5‰. This ^15^N-enrichment may suggest that some cattle consumed ^15^N-enriched plants, possibly due to fertilization with manure. However, the impact of animal dung on plant δ^15^N values is highly variable, depending on factors such as manuring frequency and type of fertilizer used [77,78,93]. High δ^15^N values could indicate that cattle had access to grazing in manured fields or consumption of agricultural surpluses, such as crop stubble. These practices have been documented in various Neolithic contexts across Europe [93,94] and the Iberian Peninsula [6–9]. Additionally, pathologies documented in the distal extremities at the Niuet site, associated with repetitive mechanical activities like animal traction, could suggest that some cattle were used in agricultural or traction tasks, providing them access to crop fields [45]. The difference in δ^15^N values between the Mas d’Is and Niuet sites, and the documented cattle bone pathologies in Niuet site, suggest a progressive intensification of cattle management over time. It should be noted that the individuals with C_4_ isotopic signatures do not show ^15^N-enriched in Mas d’Is or Niuet sites.

The isotopic signatures from this study suggests a bimodal distribution of the cattle herds over the landscape. On the one hand we have some cattle that had access to pastures in marshes or along riverbanks, such as the Gaianes Lagoon area, where they could consume plants with a C_4_ isotopic signature. Floodplain pastures offer an abundance of fresh vegetation for much of the year, allowing cattle to optimize the available natural resources. Additionally, cattle can endure prolonged periods in flooded areas due to various physiological adaptations, such as thick skin on their limbs that protects against moisture and infections, and strong hooves that enable them to withstand wet and muddy areas [95]. This access to flood zones by cattle grazing has been documented in several historical and contemporary areas such as the Camargue in France [96], Eastern Europe [97], South Sudan [98], or Australia [99] amongst many others. In the Iberian Peninsula, access by cattle to flooded sites has not yet been documented. Other isotopic data from Neolithic sites available that show a similar pattern of differential C_3_ and C_4_ isotopic signatures in cattle belong to the sites of La Vital, located at the mouth of the Serpis River and surrounded by marshes, and the site of Costamar, located on the coastal plain and also surrounded by marshes [88,89]. These results may have indicated cattle access to flood zones as well. The low δ^15^N values would reinforce this hypothesis, since they are associated with extensive husbandry practices, or even with wild, feral or semidomesticated animals. Regarding the status of animals, biometric analyses from Mas d’Is and Niuet have characterized individuals in their domestic form, but, with the data available we are unable to differentiate feral from hybrid animals [45].

Meanwhile, on the other hand, the rest of the cattle at Mas d’Is and Niuet sites grazed in pasture areas dominated by C_3_ plants, with similar grazing patterns than pig and caprine in both sites. The ^15^N-enrichment observed in some of these cattle could indicate varying levels of management intensity, with high δ^15^N values correlating with intensification of agricultural practices. Studies by García Atiénzar [36], Bernabeu Aubán [100], McClure et al. [38] have indicated that the Neolithic settlements in the Serpis Valley were strategically located in fertile valley bottoms surrounded by rich agricultural soils. These locations suggest the development of intensive agriculture with a hoe in fields close to the inhabited centres [36–38]. Therefore, at Mas d’Is and Niuet sites, specimens with ^15^N-enrichment C_3_ might have been household managed, relying on the fields nearby the settlements for access to resources or agricultural by-products.

### Husbandry practices in an environmental and socioeconomic context

Isotopic analyses of the Mas d’Is and Niuet sites reveal different strategies in animal management and landscape use strategies during the Neolithic in the Serpis Valley. The results suggest compartmentalized land use, with complementary exploitation of agricultural zones, grazing areas, and lacustrine ecosystems, in response to environmental conditions and socioeconomic transformations.

By the mid-6th millennium cal. BCE, the Serpis Valley saw effective occupation, characterized by valley bottom settlements such as the Mas d’Is site, alongside cave and shelter occupations [36–38]. According to García Atiénzar [36], low demographic density characterized these communities, with concentration of open-air settlements in particular points of the territory and focusing on small-scale cultivation and livestock management. The riverine areas, together with marshes and lakes of the valley, may have played a crucial role in these systems, offering available grazing areas for livestock and influencing settlement and mobility patterns. Furthermore, the potential for communal exploitation of these natural resources, alongside other surrounding environments, cannot be overlooked [36]. By the 4th millennium cal BCE, the process of intensification in the Serpis Valley continued, marked by new settlements in previously unoccupied areas, such as Niuet site. This change marked a transition to an aggregated settlement system with a reduction in cave-pens [40], surrounded by fields of crops and delimited by ditch [36], and the presence of negative structures, interpreted as fodder storage [44,100]. By the end of the 4th millennium cal. BCE, a significant shift occurred, marked by a reduction in the variety of cultivated cereals, indicating a trend towards relative specialization in agricultural practices [40]. Additionally, the intensification of cattle management may be associated with the occupation of the valley bottoms, where favourable conditions such as expansive plains, access to water, and abundant high-quality herbaceous grasses provided ideal grazing environments for herds. These areas, particularly along river and lake valleys, provided abundant grazing opportunities, facilitating the expansion of cattle breeding and its integration into the broader economic system [45]. Isotopic analyses of cattle, specially δ^15^N-enrichment, have revealed an intensification of management practices, further corroborated by the bone pathologies observed at the Niuet site.

The evolution and intensification of farming practices in the Serpis Valley reflect the adaptability of Neolithic communities to environmental conditions, such as topography, the availability of key resources like water, and the socioeconomic changes [101,102]. There are several historical examples of small agrarian communities in the Middle Ages and early modern period that often divided their land into two blocks: areas close to the villages, easier to work and control, because they are nearby and tend to be the flat and more fertile, tend to become the exclusive property of the family that works each plot (what capitalism has termed private property) [103–106]. Mountain, hilly or marshy areas, hard to access or to defend against interlopers, but with key resources for specific moments of the year (i.e., summer pastures), have often been managed by the entire community as commons [107]. This dynamic reflects a complex interaction between the needs of the community and households, environmental characteristics, and management capabilities, and in some ways, could coevolve through mutually beneficial interactions [99,108].

In the context of Neolithic pastoralism, property theory can offer a valuable framework for understanding territorial management practices. This theory emphasizes two key aspects: a) the characteristics of the natural resources (including their productive cycle, influenced by seasonality, topography, and water availability), and b) the characteristics of the managerial institutions developed by human communities to control these resources, influenced by human population density and competition with other groups [109]. In this framework, property is defined by the capacity to include or exclude users from access to specific resources [108]. Private property implies that an individual or household has the capacity to control and enforce a claim over a specific resource. Common property implies that a group of families exert such a claim. Open access signifies, nobody is interested or has been able to establish control over something, and that everybody has access to a given resource [110,111]. In the Serpis Valley, as isotopic results suggest, marshes or riverine areas may have played a key role in land management during the Neolithic. Marshes, characterized by difficult mobility, potential zoonosis, and/or frequent flooding, have historically been challenging environments for human settlement, making them poor candidates for the development of private property [112,113]. These complexities often led to their management as common property or their transformation into open-access resources [114,115].

Within this context, isotopic data from the Mas d’Is and Niuet sites indicate that such marginal areas also contributed significantly to husbandry practices. These marshes or riverine areas would have provided fresh fodder for much of the year and could support animals that were primarily exploited for meat in the case of cattle. Meanwhile, agricultural areas near settlements offered additional grazing opportunities, benefiting those individuals who were used for traction work. These specimens could be associated with those with C_3_ signals and higher ^15^N values. The ethnography of pastoralism identifies, plenty of cases in which animals circulate through all the areas of a given landscape and marshes are used, depending on the latitude, as winter or dry season pastures [116,117]. In other words, the resources are browsed considering the seasonal productivity of each ecological patch and niche. In this situation, however, the C4 signature is so high that the intermittent use of pastures in marshes and dryland, seems unlikely. The exclusive presence of nitrogen in the C3 animals works as well, against this possibility. To fully discard seasonal alternance between C3 and C4 areas a carbon and oxygen isotopic analysis should be implemented. This dual approach to animal management—combining a settlement-based individuals engaged in agricultural work with a more isolated individuals grazing in marshlands—reflects the emergence of a complex system of resource use. By the 4th millennium cal BCE, archaeozoological data suggest an intensification of livestock specialization, with increased production and the accumulation of surpluses, underscoring the growing economic importance of livestock, specially cattle management, and the specialization of these practices.

In addition to the direct use of marshlands or riverine areas, social and economic factors likely shaped how different groups within the Neolithic communities accessed and managed land [101]. Isotopic results reveal two different cattle feeding patterns at each site analysed, suggesting that socioeconomic implications could have influenced access to different grazing areas. Ethnographic examples suggest that differential access to marginal areas could be related to identity markers such as economic status, kinship, age, gender, or ethnicity [118–120].

Alternatively, comparative ethnographic and archaeological analysis suggest that in some inhospitable areas, escaped domestic animals may become feral and adapt to suboptimal environments [121,122]. Neighbouring communities, not necessarily involved in the management of these relatively dangerous environments might not interfere, besides the occasional capture when needed, with this rewilding process. In some cases, these feral animals have become intrinsic parts of the functionality of these environments [123,124] and become an additional source of meat when needed [125]. The C4 cattle individuals could indicate the presence of these semi-wild herds, with little interaction with nitrogen-rich or C3 plants. However, as abovementioned, only the domesticated status has been documented.

The diachronic evolution of land management strategies in the Serpis Valley highlights a gradual shift from small-scale, localized agricultural practices to more complex and integrated systems. The integration of marshes and other difficult-to-access landscapes into the broader land management system illustrates a sophisticated approach to resource use, where communal property regimes and seasonal variations in resource availability played a crucial role. From the 6th to the 3rd millennium cal BCE, the growing complexity of settlement patterns, resource use, and livestock management reflects the increasing integration of agriculture and pastoralism. By the 4th millennium cal BCE, as population densities grew and economic practices intensified, driven by both environmental pressures and social changes, underscores the resilience and adaptability of Neolithic communities in the Serpis Valley, who shaped their landscapes and economies to meet the demands of both subsistence and surplus production, ensuring the sustainability of their agricultural and pastoral practices.

## Conclusion

The variability in δ^13^C and δ^15^N values at the Mas d’Is and Niuet sites reveals a complex interplay between environmental conditions, feeding strategies, and animal management practices during the Neolithic in the Serpis Valley. The diverse δ^13^C and δ^15^N values indicate that cattle had access to a variety of spatially segregated plant resources, suggesting a nuanced approach to livestock management. Notably, the differential presence of C_3_ and C_4_ plants in the diets of these animals reflects the availability of different grazing environments, including marshlands, which may have been managed as communal or open access resources. The high nitrogen values in some cattle suggest access to fertilized pastures or agricultural by-products, highlighting the integration of animal husbandry with early agricultural practices. The fact that these higher nitrogen values coincide with C_3_ specimens seems to provide further evidence of a two-prone approach to cattle breeding: a) a settlement-based herd, part of a process of a relative agricultural intensification, grazing on the fields and pastures nearby the settlements and involved in the agricultural works, and b) a more isolated herd, mostly located and feeding in marshland and, apparently not involved with the larger agricultural system developing in the area. Furthermore, variations in the diets of caprines and pigs point to differing management strategies, with implications for social dynamics and economic organization within these Neolithic communities. The isotopic evidence extracted from the zooarchaeological remains combined with a study of their morphological incidences, supports the idea of a diversified landscape management system in the Serpis Valley, where resource availability and social structure influenced grazing practices and herd use. Furthermore, between the 6th and the beginning of the 5th millennium BCE, there was a significant shift in the strategies for occupying and exploiting territory, which may imply a new approach to animal management. The data become increasingly complete in the 4th and 3rd millennia cal BCE, revealing a certain cattle specialization and a substantial rise in production and surplus accumulated by the household units. This consolidation of husbandry practices in the Neolithic underscores the dynamic relationship between communities and their environments, reflecting an adaptive response to changing economic and social conditions.

## Supporting information

S1 Table Taxonomic representation of animal remains recovered in Phase 5 and Phase 6 of the Mas d’Is site (Penàguila, Alicante). NISP = Number of Identified Specimens; MNI = Minimum Number of Individuals.
(DOCX)

S2 Table Taxonomic representation of charcoals remains in Phase 5 and Phase 6 of the Mas d’Is site (Penàguila, Alicante). (DOCX)

## References

[1] Balasse M, Tornero C, Evin A, Tresset A, Frémondeau D, Cucchi T, et al. The place of domestic pig in the Romanian Gumelniţa (5th millennium BC): insights from the zooarchaeological analysis of Borduşani-Popină, Hârşova-tell and Vităneşti Măgurice. In Social Dimensions of Food in the Prehistory of the Eastern Balkans and Neighbouring Areas, Heidelberg (Germany); 2015.

[2] Balasse M, Evin A, Tornero C, Radu V, Fiorillo D, Popovici D, et al. Wild, domestic and feral? Investigating the status of suids in the Romanian Gumelniţa (5th mil. cal BC) with biogeochemistry and geometric morphometrics. J Anthropol Archaeol. 2016;42:27–36.

[3] Madgwick R, Esposito C, Lamb AL. Farming and feasting during the Bronze Age–Iron Age transition in Britain (ca. 900–500 BCE): multi-isotope evidence for societal change. Front Environ Archaeol. 2023;2:1221581.

[4] Makarewicz CA. Extensive woodland pasturing supported Pitted Ware Complex livestock management systems: multi-stable isotope evidence from a Neolithic interaction zone. J Archaeol Sci. 2023;158:105689.

[5] Zavodny E, McClure SB, Culleton BJ, Podrug E, Kennett DJ. Neolithic animal management practices and stable isotope studies in the Adriatic. Environ Archaeol. 2014;19(3):184–95.

[6] Navarrete V, Colonese AC, Tornero C, Antolin F, Von Tersch M, Eulàlia Subirà M, et al. Feeding management strategies among the early Neolithic pigs in the NE of the Iberian Peninsula. Int J Osteoarchaeol. 2017;27(5):839–52.

[7] Navarrete V, Viñerta A, Clemente-Conte I, Gassiot E, Rey Lanaspa J, Saña M. Early husbandry practices in highland areas during the Neolithic: the case of Coro Trasito cave (Huesca, Spain). Front Environ Archaeol. 2023;2:1309907.

[8] Navarrete V, Cardoso JL, Dias CB, Detry C, Curto A, Waterman AJ, et al. Estratégias alimentares dos animais domésticos do povoado de Leceia (Oeiras, Portugal) durante a transição do 4. o para o 3. o milénio aC: uma abordagem a partir dos isótopos estáveis. Estudos Arqueológicos de Oeiras. 2024;34:233–48.

[9] Navarrete V, Sierra A, Alcàntara R, Camalich MD, Martín-Socas D, Saña M. Integrative approaches to the study of animal management practices during the Neolithic of South Iberian Peninsula: the case of El Toro cave (Antequera, Málaga, Spain). Archaeol Anthropol Sci. 2024;16(1):21.

[10] Sierra A. Domesticación y primeras prácticas ganaderas en los Pirineos centrales. Una aproximación desde la arqueozoología y la biogeoquímica. Universidad de Zaragoza; 2020.

[11] Villalba-Mouco V, Utrilla P, Laborda R, Lorenzo JI, Martínez-Labarga C, Salazar-García DC. Reconstruction of human subsistence and husbandry strategies from the Iberian Early Neolithic: A stable isotope approach. Am J Phys Anthropol. 2018;167(2):257–71. doi: 10.1002/ajpa.23622 30129180

[12] Bernabeu Aubán J, Orozco T, Diez A, Gómez-Puche M, Molina FJ. Mas d’Is (Penàguila, Alicante): aldeas y recintos monumentales del Neolítico inicial en el valle del Serpis. Trabajos de prehistoria. 2003;60(2):39–59.

[13] Fairén Jiménez S. El paisaje de la neolitización: arte rupestre, poblamiento y mundo funerario en las comarcas centro-meridionales valencianas (Colección Arqueología; vol. 14). Alicante: Publicaciones de la Universidad de Alicante, Universidad de Alicante; 2006. p. 332.

[14] García-Puchol O, Aura JE. El abric de la Falguera (Alcoi, Alacant): 8.000 años de ocupación humana en la cabecera del río de Alcoi. Alcoy: Ayuntamiento de Alcoy; 2006.

[15] Martí B. La cova de l’or (Beniarrés, Alicante). SAGVNTVM Papeles Lab Arqueol Valencia Extra. 2011;12:183–6.

[16] Pascual Benito JL. L’abric de l’Escurrupènia (Cocentaina, Alacant). Enterrament múltiple de cremació del Neolític IIB. APL. 1990;XX:167–86.

[17] Bernabeu Aubán J. El III milenio aC en el País Valenciano: los poblados de Jovades (Cocentaina, Alacant) y Arenal de la Costa (Ontinyent, València). SAGVNTVM Papeles del Laboratorio de Arqueología de Valencia. 1993;26:9–179.

[18] Jover Maestre J. La Torreta, el Monastil (Elda, Alicante): del IV al III milenio aC en la cuenca del río Vinalopó (Memorias Excavaciones Arqueológicas, vol. 5). 2010.

[19] García-Puchol O, Molina Balaguer L, Cotino Villa F, Pascual Benito JL, Orozco Köhler T, Pardo Gordó S, et al. Hábitat, marco radiométrico y producción artesanal durante el final del Neolítico y el Horizonte Campaniforme en el corredor de Montesa (Valencia). Los yacimientos de Quintaret y Corcot. Archivo de Prehistoria Levantina. 2014;30:159–211.

[20] Pérez-Jordà G, Bernabeu J, Carrión Y, García O, Molina L, Gómez M. La Vital (Gandía, Valencia). Vida y muerte en la desembocadura del Serpis durante el III y el I milenio AC [Internet] (Trabajos Varios del SIP; vol. 113). 2011. Available from: http://www.museuprehistoriavalencia.es/web_mupreva_dedalo/publicaciones/144/es

[21] Bernabeu Aubán J, Pascual Benito JL, Orozco Köhler T, Badal Garcia E, Fumanal García MP, García-Puchol O. Niuet (L’Alqueria d’Asnar). Poblado del III Milenio aC. Recerques del Museu d’Alcoi. 1994;3:9–74.

[22] Bernabeu Aubán J, Pérez G, Molina L. La vital, Gandia (València). Un assentament del primer campaniforme a la desembocadura del Serpis. Cota cero: revista d’arqueologia i ciència. 2006;14–6.

[23] Bernabeu Aubán J, Molina Balaguer L, Orozco-Köhler T, Diez Castillo A, Barton C. Early neolithic at the Serpis Valley, Alicante, Spain. 2008:53–9.

[24] Diez-Castillo A, Barton CM, La Roca-Cervigón N, Bernabeu-Aubán J. Landscape socioecology in the Serpis Valley (10,000–4000 BP). Identifying settlement patterns and territories. In: Layers of Perception – CAA 2008, 2008. p. 1–8.

[25] García-Puchol O. Cap a la complexitat económica social i ritual: el Neolític final/Calcolític. In: Museu Arqueològic d’Alcoi (1945-2020): 75 anys cuidant el nostre patrimoni. Alcoi: Ayuntamiento de Alcoy/Ajuntament d’Alcoi; 2020. p. 89–95.

[26] Molina Ll, Bernabeu J, Orozco T. El mas d’Is (Penàguila, Alicante). SAGVNTVM Papeles Lab Arqueol Valencia. 2011;12:179–82.

[27] Torregrosa P, Jover FJ, López E. Benàmer (Muro d’Alcoi, Alicante). Mesolíticos y neolíticos en las tierras meridionales valencianas. Serie de Trabajos Varios del SIP. 2011;112:5–11.

[28] Bernabeu Aubán J, Molina Ll, editors. La Cova de les Cendres (Moraira-Teulada, Alicante). Serie Mayor; 2009. (Serie Mayor-Estudis).

[29] Esquembre MA, Soler JD, Jover FJ, Molina FJ, Luján A, Fernández J, et al. El yacimiento neolítico del Barranquet de Oliva (Valencia). In: IV Congreso del Neolítico Peninsular. 2006;183–90.

[30] Soler J, Gómez-Pérez O, García-Atiénzar G, Roca de Togores Muñoz C. Sobre el primer horizonte neolítico en la Cova d’En Pardo (Planes, Alicante): su evaluación desde el registro cerámico. 2011.

[31] Soler J, Roca C, et al. Progresos en la investigación del fenómeno de inhumación múltiple en La Marina Alta (Alicante). A propósito de los trabajos desarrollados en la Cova del Randero de Pedreguer y en la Cova del Barranc del Migdia de Xàbia. Del Neolític a l’Edat de Bronze en el Meditarrani occidental: estudis en homenatge a Bernat Martí Oliver TV SIP. 2016;119.

[32] Pascual Benito J, Barberà M, Ribera A. El Camí de Missena (La Pobla del Duc): un interesante yacimiento del III milenio en el País Valenciano. In Santander: Servicio de Publicaciones; 2005. p. 803–14.

[33] Gómez Puche M, Diez Castillo A, Verdasco Cebrián C, García Borja P, McClure SB, López Gila MD, et al. El yacimiento de Colata (Montaverner, Valencia) y los “poblados de silos” del IV milenio en las comarcas centro-meridionales del País Valenciano. Recerques del Museu d’Alcoi. 2004;13:53–128.

[34] McClure SB, Welker M. Farming with animals: Domesticated animals and taxonomic diversity in the cardial Neolithic of the Western Mediterranean. In: García-Puchol O, Salazar-García DC, editors. Times of Neolithic transition along the Western Mediterranean. Springer; 2017. p. 221–50.

[35] Pérez-Ripoll M. La explotación ganadera durante el III milenio a. C. en la Península Ibérica. SAGVNTVM Papeles Lab Arqueol Valencia-Extra. 1999;2:95–103.

[36] García-Atiénzar G. Territorio Neolítico. Las primeras comunidades campesinas en la fachada oriental de la península Ibérica (ca. 5600-2800 cal BC). Oxford; 2009. (BAR Internacional Series).

[37] Bernabeu Aubán J. Origen y consolidación de las sociedades agrícolas. El País Valenciano entre el Neolítico y la Edad del Bronce. Jornades d’Arqueologia Valenciana. 1995;37–60.

[38] McClure SB, Barton CM, Jochim MA. Human behavioral ecology and climate change during the transition to agriculture in Valencia, eastern Spain. J Anthropol Res. 2009;65(2):253–69.

[39] Pérez-Jordà G, Peña-Chocarro L, Mateos JM, Zapata L. Evidence for early crop management practices in the Western Mediterranean: latest data, new developments and future perspectives. In: Times of Neolithic transition along the Western Mediterranean. 2017. p. 171–97.

[40] Pérez-Jordà G, Peña-Chocarro L. Agricultural production between the 6th and the 3rd millennium cal BC in the central part of the Valencia region (Spain). In: Groot M, Lentjes D, Zeiler J, editors. Barely Surviving or More than Enough? The Environmental Archaeology of Subsistence, Specialisation and Surplus Food Production. Leiden, The Netherlands: Sidestone Press; 2013. p. 81–100.

[41] Iborra Eres MP, Martínez-Valle R. La fauna de los niveles neolíticos de la Cova de les Cendres. In: La Cova de Les Cendres:(Moraira-Teulada, Alicante). Museo Arqueológico de Alicante-MARQ; 2009. p. 149–62.

[42] Pérez-Ripoll M. La fauna de vertebrados. In: Cova de l’Or (Beniarrés-Alicante): Vol II. València: Museu de Prehistòria de València; 1980. p. 193–255. (Trabajos Varios del SIP; vol. II).

[43] Boessneck J, von den Driesch A. Tierknochenfunde aus der Südspanischen Hohlen. Studien uber Tierknochenfunde von der Iberischen Halbinsel. 1980;7:1–83.

[44] Martínez-Valle R. La fauna de vertebrados, en J. Bernabeu (dir.), El III milenio AC en el País Valenciano. Los poblados de Jovades (Cocentaina, Alacant) y Arenal de la Costa (Ontinyent, València). SAGVNTVM Papeles Lab Arqueol Valencia; 1993.

[45] Navarrete V, Escribá Ruiz P, García-Puchol O, Bernabeu Auban J, Pérez-Ripoll M. Caracterización de las prácticas ganaderas del yacimiento de Niuet (l’Alqueria d’Asnar, Alicante) en el contexto de los asentamientos del IV al III milenio a.C. en el este de la Península Ibérica. Lucentvm. 2025;44:55–69.

[46] Iborra Eres MP, López Gila MD. La ganadería y la caza. In: La Vital (Gandia, Valencia): vida y muerte en la desembocadura del Serpis durante el III y el I milenio aC. SIP-Diputación de valencia. Museu de Prehistòria de València; 2011. p. 105–20. (Trabajos Varios del SIP).

[47] Sánchez Goñi MF, Harrison SP. Vegetation and climate in the Iberian Peninsula during the Last Glacial-Interglacial Transition: A review of the palynological data. Quaternary Sci Rev. 2010;29(1–2):116–36.

[48] Badal E, Bernabeu J, Vernet JL. Vegetation changes and human action from the Neolithic to the bronze age (7000–4000 BP) in Alicante, Spain, based on charcoal analysis. Vegetat History Archaeobotany. 1994;3:155–66.

[49] Badal E, Martí Oliver B, Pérez Ripoll M. From agricultural to pastoral use: changes in Neolithic landscape at Cova de l’Or (Alicante, Spain)”. In: Badal et al., editors. Wood and charcoal. Evidence for human and natural History. Universitat de València (Saguntum Extra-13). València; 2012. p. 75–84.

[50] García A, García R. La Albufera de Gaianes: Un humedal en el corazón de la comarca del Comtat. Boletín de la Sociedad de Historia Natural de Valencia. 2020;50:83–96.

[51] Bernabeu Aubán J, Diez Castillo A, Orozco-Köhler T. Campañas de excavación recientes en el yacimiento del Mas d’ls (Penàguila, Alacant). Marq arqueología y museos. 2014;1:183–8.

[52] Bernabeu J, Jiménez-Puerto J, Escribá P, Pardo-Gordó S. C14 y poblamiento en las comarcas centro-meridionales del País Valenciano (c. 7000-1500 cal BC). Recerques del Museu d’Alcoi. 2018;27:35–48.

[53] Bernabeu Aubán J, Pardo-Gordó S. La Impressa en la península Ibérica:¿espejismo o realidad? Una reflexión a partir del binomio radiocarbono-cerámica. In: Contextualizando la cerámica Impressa: horizontes culturales en la península ibérica. 2020. p. 47–57.

[54] Carrión Y. La vegetación mediterránea y atlántica de la Península Ibérica. Nuevas secuencias antracológicas. SIP-Diputación Provincial de Valencia. Valencia; 2005. p. 314 (Trabajos Varios del SIP; vol. 104).

[55] Reimer P, Austin W, Bard E, Bayliss A, Blackwell PG, Bronk Ramsey C, et al. The IntCal20 Northern Hemisphere radiocarbon age calibration curve (0-55 cal kB). Radiocarbon [Internet]. 2020;62:725–57. Available from: http://calib.org/calib/calib.html

[56] Bronk Ramsey C. OxCal 4.4. 4 calibration program. 2021. Available from: https://c14archox.ac.uk/oxcal/OxCal.html

[57] Escribá Ruiz P. Geometría y decoración del Xúquer al Ebre del VI al V milenio cal BC. [Internet]. Servei d’Investigacions Arqueològiques i Prehistòriques. Diputació de Castelló. Castellón: Diputación de Castellón; 2023. p. 380 (Monografíes de Prehistòria i Arqueologia Castellonenques.). Available from: https://dialnet.unirioja.es/servlet/libro?codigo=931630

[58] Bernabeu Aubán J, Lozano S, Pardo-Gordó S. Iberian Neolithic Networks: The Rise and Fall of the Cardial World. Front Digital Human [Internet]. 2017 [cited 2017 May 16]; 4:01–19. Available from: http://journal.frontiersin.org/article/10.3389/fdigh.2017.00007/full

[59] Bernabeu Aubán J, Badal García E. A view of the vegetation and economic exploitation of the forest in the Late Neolithic sites of Les Jovades and Niuet (Alicante, Spain). Bulletin de la Société Botanique de France Actualités Botaniques. 1992;139(2–4):697–714.

[60] Longin R. New method of collagen extraction for radiocarbon dating. Nature. 1971;230(5291):241–2. doi: 10.1038/230241a0 4926713

[61] Hammer Ø, Harper DA. Past: paleontological statistics software package for education and data analysis. Palaeontologia Electronica. 2001;4(1):1–9.

[62] Ambrose SH, Norr L. Experimental evidence for the relationship of the carbon isotope ratios of whole diet and dietary protein to those of bone collagen and carbonate. In: Prehistoric human bone: archaeology at the molecular level. Springer; 1993. p. 1–37.

[63] Schwarcz HP, Schoeninger MJ. Stable isotope analyses in human nutritional ecology. Am J Physical Anthropol. 1991;34(S13):283–321.

[64] Sealy J, Brothwell D. Body tissue chemistry and palaeodiet. In: Pollard AM, editor. Handbook of archaeological sciences. Chichester: John Wiley and Sons; 2001. p. 269–79.

[65] Hedges RE, Clement JG, Thomas CDL, O’Connell TC. Collagen turnover in the adult femoral mid-shaft: modeled from anthropogenic radiocarbon tracer measurements. Am J Physical Anthropol. 2007;133(2):808–16. 10.1002/ajpa.20598 17405135

[66] Bocherens H, Fizet M, Mariotti A, Lange-Badré B, Vandermeersch B, Borel J, et al. Isotopic biogeochemistry (δ13C, δ15N) of fossil vertebrate collagen: implications for the study of fossil food web including Neandertal man. J Hum Evol. 1991;20:481–92.

[67] Bocherens H, Fogel ML, Tuross N, Zeder M. Trophic structure and climatic information from isotopic signatures in Pleistocene cave fauna of southern England. J Archaeol Sci. 1995;22(2):327–40.

[68] Schoeninger MJ, DeNiro MJ. Nitrogen and carbon isotopic composition of bone collagen from marine and terrestrial animals. Geochimica et Cosmochimica Acta. 1984;48(4):625–39. doi: 10.1016/0016-7037(84)90091-7

[69] Bender MM. Variations in the 13C/12C ratios of plants in relation to the pathway of photosynthetic carbon dioxide fixation. Phytochemistry. 1971;10(6):1239–44.

[70] Farquhar GD, Ehleringer JR, Hubick KT, et al. Carbon isotope discrimination and photosynthesis. Annual Rev Plant Physiol Plant Molecular Biol. 1989;40(1):503–37.

[71] Körner C, Farquhar GD, Wong SC. Carbon isotope discrimination by plants follows latitudinal and altitudinal trends. Oecologia. 1991;88(1):30–40. doi: 10.1007/BF00328400 28312728

[72] Tieszen LL. Natural variations in the carbon isotope values of plants: implications for archaeology, ecology, and paleoecology. J Archaeol Sci. 1991;18(3):227–48.

[73] Friedli H, Lötscher H, Oeschger H, Siegenthaler U, Stauffer B. Ice core record of the 13C/12C ratio of atmospheric CO2 in the past two centuries. Nature. 1986;324(6094):237–8.

[74] Hellevang H, Aagaard P. Constraints on natural global atmospheric CO2 fluxes from 1860 to 2010 using a simplified explicit forward model. Sci Rep. 2015;5:17352. doi: 10.1038/srep17352 26611741 PMC4679175

[75] O’Leary MH. Carbon isotope fractionation in plants. Phytochemistry. 1981;20(4):553–67.

[76] O’Connell TC, Kneale CJ, Tasevska N, Kuhnle GGC. The diet-body offset in human nitrogen isotopic values: a controlled dietary study. Am J Phys Anthropol. 2012;149(3):426–34. doi: 10.1002/ajpa.22140 23042579 PMC3483624

[77] Bogaard A, Heaton TH, Poulton P, Merbach I. The impact of manuring on nitrogen isotope ratios in cereals: archaeological implications for reconstruction of diet and crop management practices. J Archaeol Sci. 2007;34(3):335–43.

[78] Szpak P. Complexities of nitrogen isotope biogeochemistry in plant-soil systems: implications for the study of ancient agricultural and animal management practices. Front Plant Sci. 2014;5:288. doi: 10.3389/fpls.2014.00288 25002865 PMC4066317

[79] DeNiro MJ. Postmortem preservation and alteration of in vivo bone collagen isotope ratios in relation to palaeodietary reconstruction. Nature. 1985;317(6040):806–9.

[80] Van Klinken GJ. Bone collagen quality indicators for palaeodietary and radiocarbon measurements. J Archaeol Sci. 1999;26(6):687–95.

[81] Ambrose SH. Preparation and characterization of bone and tooth collagen for isotopic analysis. J Archaeol Sci. 1990;17(4):431–51.

[82] Heaton TH. Spatial, species, and temporal variations in the 13c/12c ratios of C3 plants: implications for palaeodiet studies. J Archaeol Sci. 1999;26(6):637–49.

[83] Hartman G, Danin A. Isotopic values of plants in relation to water availability in the Eastern Mediterranean region. Oecologia. 2010;162(4):837–52. doi: 10.1007/s00442-009-1514-7 19956974 PMC2841277

[84] Smedley MP, Dawson TE, Comstock JP, Donovan LA, Sherrill DE, Cook CS, et al. Seasonal carbon isotope discrimination in a grassland community. Oecologia. 1991;85(3):314–20. doi: 10.1007/BF00320605 28312034

[85] Pyankov VI, Ziegler H, Akhani H, Deigele C, Luettge U. European plants with C4 photosynthesis: geographical and taxonomic distribution and relations to climate parameters. Botanical J Linnean Soc. 2010;163(3):283–304.

[86] Morris J, McGowan S. C4 photosynthesis: a key to understanding wetland plant dynamics. Wetlands Ecol Manag. 2004;11(2):91–9.

[87] Sage RF. The evolution of C4 photosynthesis. New Phytol. 2004;161(2):341–70. doi: 10.1111/j.1469-8137.2004.00974.x 33873498

[88] Salazar-García DC. Estudio de la dieta en la población neolítica de Costamar. Resultados preliminares de análisis de isótopos estables de carbono y nitrógeno. Torre la Sal (Ribera de Cabanes, Castellón) Evolución del paisaje antrópico desde la prehistoria hasta el medioevo. 2009; 8:411–8.

[89] Salazar-García DC. Aproximación a la dieta de la población calcolítica de La Vital a través del análisis de isótopos estables del Carbono y del Nitrógeno sobre restos óseos. In: Pérez Jordà G, Bernabeu Aubán J, Carrión Marco Y, García Puchol O, Molina Balaguer L, Gómez Puche M, editors. La Vital (Gandia, Valencia): Vida y muerte en la desembocadura del Serpis durante el III y el I milenio aC. Museu de Prehistòria de València; 2011. p. 139–43. (Trabajos Varios del SIP).

[90] Hamilton J, Thomas R. Pannage, pulses and pigs: isotopic and zooarchaeological evidence for changing pig management practices in later medieval England. Medieval Archaeol. 2012;56(1):234–59.

[91] Madgwick R, Mulville J, Stevens RE. Diversity in foddering strategy and herd management in late Bronze Age Britain: an isotopic investigation of pigs and other fauna from two midden sites. Environmental Archaeol. 2012;17:126–40.

[92] Mellor DJ, Stafford KJ. Animal welfare implications of neonatal mortality and morbidity in farm animals. Vet J. 2004;168(2):118–33. doi: 10.1016/j.tvjl.2003.08.004 15301760

[93] Fraser RA, Bogaard A, Heaton T, Charles M, Jones G, Christensen BT. Manuring and stable nitrogen isotope ratios in cereals and pulses: towards a new archaeobotanical approach to the inference of land use and dietary practices. J Archaeol Sci. 2011;38(10):2790–804.

[94] Bogaard A, Fraser R, Heaton THE, Wallace M, Vaiglova P, Charles M, et al. Crop manuring and intensive land management by Europe’s first farmers. Proc Natl Acad Sci U S A. 2013;110(31):12589–94. doi: 10.1073/pnas.1305918110 23858458 PMC3732975

[95] Decker JE, Rowan TN. Cattle losing adaptations to environmental stressors, MU Researchers Find. University of Missouri, College of Agriculture, Food and Natural Resources; 2021.

[96] Crouzat E, Mazzarella S. Understanding the ecological roles of livestock in Mediterranean wetlands: insights from the Camargue. Wetlands Ecol Manag. 2011;29(5):629–45.

[97] Biró M, Molnár Z, Öllerer K, Lengyel A, Ulicsni V, Szabados K, et al. Conservation and herding co-benefit from traditional extensive wetland grazing. Agriculture Ecosyst Environ. 2020;300:106983.

[98] Mirski P. Space use by semi‐free‐ranging cows on wetlands and its implication as a conservation management tool. Restoration Ecol. 2022;30(3):e13533.

[99] Morris K, Reich P. Understanding the relationship between livestock grazing and wetland condition. Arthur Rylah Institute for Environmental Research Technical Report Series. 2013; 252.

[100] Bernabeu Aubán J, Badal E. Imagen de la vegetación y utilización económica del bosque en los asentamientos neolíticos de Jovades y Niuet (Alicante). Archivo de Prehistoria Levantina. 1990;20.

[101] Bernabeu Aubán J, Balaguer LM, Castillo AD, Köhler TO. Inequalities and power. Three millennia of prehistory in Mediterranean Spain (5600–2000cal BC). In Social Inequality in Iberian Late Prehistory. BAR International Series. Oxford: Archaeopress; 2006. p. 97–116.

[102] García-Puchol O, Molina Balaguer L, Cotino Villa F, Pascual Benito JL, Orozco Köhler T, Pardo Gordó S, et al. Hábitat, marco radiométrico y producción artesanal durante el final del Neolítico y el Horizonte Campaniforme en el corredor de Montesa (Valencia). Los yacimientos de Quintaret y Corcot. Archivo de Prehistoria Levantin. 2014;30:159–211.

[103] Beltràn O, Vaccaro l. Los comunales en el Pirineo central. Idealizando el pasado y reelaborando el presente. Revista de Antropología Social. 2017;26(2):235–57.

[104] Guadilla-Sáez S, Pardo-de-Santayana M, Reyes-García V. Forest commons, traditional community ownership and ecological consequences: insights from Spain. Forest Policy Econ. 2020;112:102107.

[105] Lana JM, Iriarte-Goñi I. Commons and the legacy of the past. Regulation and uses of common lands in twentieth century Spain. Int J Commons. 2015;9(2):510–32.

[106] Serrano Alvarez JA. When the enemy is the state: common lands management in northwest Spain (1850–1936). Int J Commons. 2014;8(1):107–33.

[107] Beltràn O, Vaccaro I. Between communal herding and state parcellation: The conflicting territorialities of the Spanish Pyrenees. In: Dawson C, Zanotti L, Vaccaro I, editors. Negotiating territoriality: Spatial dialogues between state and tradition. New York: Routledge; 2014. p. 21–35.

[108] Jiménez GB. Bondades ecológicas del búfalo de agua: camino hacia la certificación. Tecnología en Marcha. 2011;24(5):82–8.

[109] Bromley D. Making the Commons Work: Theory, Practice, and Policy. In: Bromley D, editor. Southern Economic Journal. ICS-Press. San Francisco: Southern Economic Association; 1992.

[110] Ribot JC, Peluso NL. A theory of access. Rural Sociol. 2003;68(2):153–81.

[111] Vaccaro I, Beltran O. What Do We Mean by “the Commons?” An Examination of Conceptual Blurring Over Time. Hum Ecol. 2019;47(3):331–40. doi: 10.1007/s10745-019-00081-z

[112] Dyson‐Hudson R, Smith EA. Human territoriality: an ecological reassessment. American Anthropol. 1978;80(1):21–41.

[113] Krishnan S, Pastore CL, Temple S, editors. Unruly environments. RCC; 2015.

[114] Agrawal A. Sustainable Governance of Common-Pool Resources: Context, Methods, and Politics. Annu Rev Anthropol. 2003;32(1):243–62. doi: 10.1146/annurev.anthro.32.061002.093112

[115] Ostrom E. Governing the commons: The evolution of institutions for collective action. Cambridge University Press; 1990.

[116] Fynn RW, Murray-Hudson M, Dhliwayo M, Scholte P. African wetlands and their seasonal use by wild and domestic herbivores. Wetlands Ecol Manag. 2015;23:559–81.

[117] Tamou C, de Boer IJM, Ripoll-Bosch R, Oosting SJ. Traditional ecological knowledge underlying herding decisions of pastoralists. Animal. 2018;12(4):831–43. doi: 10.1017/S1751731117002130 28849752

[118] Spencer P. The pastoral continuum: The marginalization of tradition in East Africa. Clarendon Press; 1998.

[119] Ensminger J, Rutten A. The political economy of changing property rights: dismantling a pastoral common. American Ethnol. 1991;18(4):683–99.

[120] Mulder MB, Fazzio I, Irons W, McElreath RL, Bowles S, Bell A, et al. Pastoralism and Wealth Inequality. Current Anthropol. 2010;51(1). 10.1086/648530 PMC2999363 21151711

[121] Gron KJ. The feral animal question: implications for recognizing Europe’s first farmers. European J Archaeol. 2023;26(4):410–25.

[122] Gron KJ, Gröcke DR, Groß D, Rowley-Conwy P, Robson HK, Montgomery J. Neolithisation through bone: Stable isotope analysis of human and faunal remains from Syltholm II, Lolland, Denmark. J Archaeol Sci. 2024;53:104384.

[123] Clancy C, Cooke F, Raw Z. Entanglement, autonomy and the co-production of landscapes: relational geographies for free-roaming ‘feral’ donkeys (Equus asinus) in a rapidly changing world. Geoforum. 2021;123:66–77.

[124] Kugler W, Broxham E. The ecological value of feral livestock populations in Europe. Overview, situation and development of a network for management of wild livestock populations, Final Report SAVE-Project, St Gallen, Switzerland. 2014.

[125] Gressier C. Going feral: Wild meat consumption and the uncanny in Melbourne, Australia. Australian J Anthropol. 2016;27(1):49–65.

