# Supplementary material for: Isotopes, herds, and landscape management practices: New insights on early farming communities in the Serpis Valley (Eastern Iberian Peninsula)
Source: PLoS One. 2025 Jun 27;20(6):e0325137. doi: 10.1371/journal.pone.0325137 (PMC12204573; doi:10.1371/journal.pone.0325137)
Supplement: S1 Table — NISP = Number of Identified Specimens; MNI = Minimum Number of Individuals. (DOCX) [file pone.0325137.s001.docx]

|  | **Phase 5** | | **Phase 6** | |
| --- | --- | --- | --- | --- |
|  | **NISP** | **MNI** | **NISP** | **MNI** |
| *Bos taurus* | 12 | 3 | 52 | 9 |
| *Capra hircus* | 1 | 1 | 2 | 1 |
| *Ovis aries* | 3 | 1 | 7 | 2 |
| *Ovis/Capra* | 7 | 2 | 28 | 3 |
| *Sus domesticus* | 15 | 3 | 2 | 1 |
| *Cervus elaphus* | 13 | 3 | 23 | 3 |
| *Oryctolagus cunniculus* | 0 | 0 | 3 | 2 |
