# Supplementary material for: Isotopes, herds, and landscape management practices: New insights on early farming communities in the Serpis Valley (Eastern Iberian Peninsula)
Source: PLoS One. 2025 Jun 27;20(6):e0325137. doi: 10.1371/journal.pone.0325137 (PMC12204573; doi:10.1371/journal.pone.0325137)
Supplement: S2 Table — (DOCX) [file pone.0325137.s002.docx]

| **TAXA** | **Phase 5** | | **Phase 6** | |
| --- | --- | --- | --- | --- |
|  | **Nº** | **%** | **Nº** | **%** |
| Monocotyledoneae | 5 | 2,6 | 0 | 0 |
| *Pinus halepensis* | 6 | 3,1 | 0 | 0 |
| *Quercus evergreen* | 183 | 94,3 | 60 | 100 |
| TOTAL | 194 | 100 | 60 | 100 |
